# Safety of Nanoclay/Spring Water Hydrogels: Assessment and Mobility of Hazardous Elements

**DOI:** 10.3390/pharmaceutics12080764

**Published:** 2020-08-12

**Authors:** Fátima García-Villén, Rita Sánchez-Espejo, Ana Borrego-Sánchez, Pilar Cerezo, Luana Perioli, César Viseras

**Affiliations:** 1 Department of Pharmacy and Pharmaceutical Technology, Faculty of Pharmacy, University of Granada, Campus of Cartuja, 18071 Granada, Spain; fgarvillen@ugr.es (F.G.-V.); mcerezo@ugr.es (P.C.); 2 Andalusian Institute of Earth Sciences, CSIC-UGR, Avenida de las Palmeras 4, 18100 Armilla, Granada, Spain; ritase@correo.ugr.es (R.S.-E.); anaborrego@iact.ugr-csic.es (A.B.-S.); 3 Department of Pharmaceutical Sciences, University of Perugia, via del Liceo 1, 06123 Perugia, Italy; luana.perioli@unipg.it

**Keywords:** heavy metal, hazardous element, element mobility, clay minerals, spring water, hydrogel, toxicity, sepiolite, palygorskite

## Abstract

The presence of impurities in medicinal products have to be controlled within safety limits from a pharmaceutical quality perspective. This matter is of special significance for those countries and regions where the directives, guidelines, or legislations, which prescribe the rules for the application of some products is quite selective or incomplete. Clay-based hydrogels are quite an example of this matter since they are topically administered, but, in some regions, they are not subjected to well-defined legal regulations. Since hydrogels establish an intimate contact with the skin, hazardous elements present in the ingredients could potentially be bioavailable and compromise their safety. The elemental composition and mobility of elements present in two hydrogels have been assessed. Sepiolite, palygorskite, and natural spring water were used as ingredients. The release of a particular element mainly depends on its position in the structure of the hydrogels, not only on its concentration in each ingredient. As a general trend, elements’ mobility reduced with time. Among the most dangerous elements, whose presence in cosmetics is strictly forbidden by European legal regulations, As and Cd were mobile, although in very low amounts (0.1 and 0.2 μg/100 g of hydrogel, respectively). That is, assuming 100% bioavailability, the studied hydrogels would be completely safe at normal doses. Although there is no sufficient evidence to confirm that their presence is detrimental to hydrogels safety, legally speaking, their mobility could hinder the authorization of these hydrogels as medicines or cosmetics. In conclusion, the present study demonstrates that hydrogels prepared with sepiolite, palygorskite, and Alicún spring water could be topically applied without major intoxication risks.

## 1. Introduction

The concentration and bioavailability of impurities such as hazardous elements in both health products and medicines is a main preformulation concern during their development. Different health care products must comply with specific normatives and guidelines, depending on the administration route, and most of the times, on the region or country in which they are written and applied. It is generally accepted that levels of elemental impurities below toxicity thresholds could be considered as safe, with diverse limits depending on the consulted normative. In Canada, natural health products that do not require a medical prescription, are included in a guide in which heavy metals (Pb, As, Cd, Hg, and Sb, among others) are banned or limited to a maximum amount, in accordance with the administration route [1]. In the USA, similar health products fail into FDA legislation, which only considers Hg as a forbidden element and limits the Pb concentration [2]. On the other hand, European cosmetic legislation is much more detailed and restrictive regarding the presence of elemental impurities [3].

Similar health products may also be considered into different categories depending on the country. The boundaries between medicinal products, natural health care products, cosmetics, and others, are not internationally normalized, even if generally accepted definitions have been achieved. In fact, the absence of clear boundaries made it necessary to address some products on a case-by-case basis. Particularly interesting is the cosmetic category, in which the presence of some ingredients, their origin, the administration route, and the scope of the product could raise doubts about their classification. A manual on the scope of the application of the cosmetics regulation EC 1223/2009 has been published by the working group on cosmetic products in order to shed some light on this matter [4]. The global cosmetics market have grown by an estimated 5.25% in 2019 [5], and, due to this continuous growth, the attention is being increasingly focused on the quality and safety of these products. Cosmetics are, according to the European Council Directive 2003/15/EC and the US Food Drug and Cosmetic Act, those products or mixtures of substances prepared and destined to be applied in different parts of the human body in order to clean, protect, maintain them in good conditions, improve their aspect, or relieve/eliminate body odors [6,7]. It has been recognized and demonstrated that, although cosmetics are intended to be applied on the surface of the body or mucous membranes, they may not remain there exclusively, since some topically applied substances may penetrate through the skin [8,9,10]. This fact is more pronounced for those cosmetics which are intended to remain at their application site for several hours or days, without subsequent rinse or wash. The European Union established a Regulation on Cosmetic Products (1223/2009) where it states that “cosmetic products should be safe under normal or reasonably foreseeable conditions of use. In particular, a risk-benefit reasoning should not justify a risk to human health” [3]. According to the European legislation, all cosmetic products should be subjected to safety assessments, taking into consideration the toxicology of all the ingredients used, as well as their chemical structure and their potential to produce local and systemic side effects.

The use of clay minerals in health care comes from prehistoric times, as reviewed in various paperwork and databases [11,12,13,14,15]. Some properties of these minerals have made them one of the most frequently used materials in pharmaceutical formulations and cosmetics, due to both their potential therapeutic activities and their useful properties as excipients. These features depend on colloidal dimensions and high surface areas of clay minerals, which give rise to optimal rheological and sorption capacities [13,16,17]. Kaolin, talc, smectites (montmorillonite and saponite), and fibrous clays (palygorskite and sepiolite) are some of the clay minerals most used in pharmacy. Hydrotherapy, and more particularly, balneotherapy, is one of the most frequent uses of clay minerals from a traditional and natural point of view. Clay minerals are used to prepare semisolid suspensions (frequently known as thermal muds or peloids) after the interposition of clays with spring waters, thus forming a nanoclay/spring water hydrogel. That is, thermal muds are semisolid, topical, natural medicinal hydrogels prepared by the interposition of organic and inorganic solids in mineral–medicinal water [11].

On the view of the uses and properties of these clay-based hydrogels, they could be considered as cosmetics with skin-care functions such as cleansing, degreasing, exfoliating, hydrating, invigorating, and firming activities [18,19,20]. These clay-based products could also be considered as medicinal products, as they have demonstrated activities against dermatological affections such as psoriasis [21,22,23], atopic dermatitis, vitiligo, eczemas, seborrhoeic dermatitis, fungal infections, or acne have also been treated by clay/spring water hydrogels [24,25,26,27,28]. Moreover, in view of the current Covid-19 worldwide state of emergency, Masiero et al. [29] have pointed out the already demonstrated positive effects of balneotherapy and thermal muds over the human immune system. Therefore, according to the guidelines of the borderline products manual [4], these formulations could fall either in the cosmetic or in the medicine category. Nonetheless, they are usually prepared in thermal stations, without a deep characterization of their therapeutical functions, activities, safety, or quality control. This unclear boundary between cosmetic and medical products showed by thermal muds, justifies the necessity of a harmonized regulation that compel for a full characterization of these products. Either way, since clay-based hydrogel formulations are intended to be applied over the skin (either health, ill, or injured skin), safety assessments should be one of the main milestones to be accomplished.

A remarkable feature of materials such as clays is the wide spectrum of mineralogical and chemical composition they have, something that is inevitable when it comes to natural products, most of the time accompanied by other naturally-associated mineral phases. Even if nanoclay/spring water hydrogels (thermal muds) are not legally considered as cosmetics or medicines and, therefore, they are not subjected to any kind of compulsory regulation; their accomplishment would highlight their quality constancy attributes and safety. The use of pharmaceutical-grade minerals in the preparation of thermal muds would guarantee the compositional safety of the gel-like system. In this regard, the chemical composition of the substances present in the formulation is crucial, since hazardous elements such as heavy metals and other elements are prohibited or limited in cosmetic products by different regulations. For instance, the ICH Harmonized Guideline for elemental impurities Q3D(R1) [30] published by the European Medicines Agency and Regulation No 1223/2009, European Parliament [3] are some of the regulations in which the discussion of this paperwork will be based. The safety assessment of the elements, such as heavy metals in cosmetics should start from the knowledge of the type and concentration of ingredients contained in the product in order to evaluate their potential intrinsic hazard [31]. The next step would include the analysis and studies of the mobility of those elements, either beneficial or harmful, in order to understand the potential biological and therapeutical effects of the formulation. This is especially important when it comes to “technically unavoidable” elements.

Recently, sepiolite and palygorskite (two inorganic excipients mainly composed of clay minerals) were mixed with natural spring water to prepare hydrogels intended for topical application. In previous studies, these excipients have demonstrated remarkable purity in terms of mineralogical composition (> 92% and > 58% of sepiolite and palygorksite richness, respectively) and quality performance [32]. Moreover, these very same hydrogels have also proved to possess wound healing effects.

The aim of this paper was to prepare, characterize, and address the elemental composition of nanoclay/spring water hydrogels made of sepiolite and palygorskite clay minerals and natural spring water. Since the present hydrogels are intended to be applied over potentially damaged or wounded skin, the mobility of their elements was also characterized. The final objective of this study was to assess the safety attributes of these formulations on the basis of the content and bioavailability of the elemental impurities.

## 2. Materials and Methods

### 2.1. Materials

Pangel S9 (PS9) and Cimsil G30 (G30) were kindly gifted by the TOLSA Group (Madrid, Spain). PS9 (d_90_ 23.9 μm) and G30 (d_90_ 49.3 μm) were mainly composed by sepiolite and palygorskite, respectively. According to their composition and properties, previously characterized by García-Villén et al. [32], they could be classified as “pharmaceutical-grade” excipients. Their corresponding pharmacopoeial denominations are “magnesium trisilicate” (PS9) and “Attapulgite” (G30) [33,34,35]. Sepiolite was present in PS9 in >92%, muscovite being the main mineralogical impurity detected. Palygorskite was present in G30 in 58%, accompanied by quartz (26%), fluorapatite (7%), smectites, and sepiolite (6%) and carbonates (3%) as associated minerals. Both PS9 and G30 excipients were dried in an oven at 40 °C for at least 48 h prior to being used in the preparation of the hydrogels. Spring water from Alicún thermal station (ALI), located in Granada (Spain) was used. ALI water is classified as hypothermal with strong mineralization [36,37].

Nanoclay/spring water hydrogels were prepared according to a process previously studied and optimized [32]. Briefly, clay minerals were mixed with ALI by means of a turbine high-speed agitator (Silverson LT, Chesham, UK), equipped with a high-traction stirrer head of square mesh, at 8000 rpm for 5 min. Samples were preserved in closed polyethylene containers, from which aliquots were sampled in order to monitor further analysis. Nanoclay/spring water hydrogels prepared had a 10% w/w of PS9 nanoclay concentration (ALIPS9) and 20% *w/w* of G30 nanoclay (ALIG30). Both of them were preserved and characterized in the same way.

### 2.2. Methods

#### 2.2.1. Elemental Composition of Pristine Ingredients

Elements present in PS9 and G30 as well as ALI water has been addressed by Inductively Coupled Plasma mass spectrometry (ICP-MS) measurements. Solid samples were prepared by acid digestion in strong acids (HNO_3_ and HF at a 3:5 ratio) inside a Teflon reactor, thus the samples were subjected to high pressure and temperature by heating in a microwave oven (Milestone ETHOS ONE, Sorisole, Italy). The quantification of the elements was done by a NexION-300 ICP-MS spectrometer (Perkin Elmer, Waltham, MA, USA) equipped with a triple cone interface and a quadrupole ion deflector using argon for plasma formation. Standard solutions of 100 and 1000 ppb were prepared for each element (Multi-Element standards, Perkin Elmer, Waltham, MA, USA), and Rh was employed as an internal standard. All standards were prepared from ICP single-element standard solutions (Merck, Darmstadt, Germany) after dilution with 10% HNO_3_. Ultrapurified water (Milli-Q^®^ grade, 18 MΩ·cm) was used during the whole experiment. The accuracy of the ICP-MS equipment used ranges between ±2 and ±5% for analyte concentrations between 50 and 5 ppm, respectively. The detection limits were <0.1 ppt for Ir and Ta; <1 ppt for Ba, Li, Cu, Mo, Sb, Sn, Ag, Au, Co, Ni, V, As, Cd, Pb, Zr, Be and Nd; < 10 ppt for Cr, Hg and Te; <1 ppb for P.

#### 2.2.2. In Vitro Release of Elemental Impurities from Hydrogels

The element mobility from nanoclay/spring water hydrogels (ALIPS9, ALIG30) was assessed by in vitro cation release studies performed in a Franz diffusion cell system FDC40020FF (BioScientific Inc., Phoenix, AZ, USA) [38]. This system is designed to recreate conditions of formulations placed over the skin and mucosa membranes. Particularly, the selected Franz diffusion cells possessed a contact area of 0.64 cm^2^ and an approximate total volume of 6.4 mL in the receptor chamber. In this study, the aim is to explore the potential number of elements that would be released by the formulation and that are potentially able to establish contact with the skin. To do so, dialysis membranes (cut-off 12–14 kDa (31.7 mm), Medicell International, London, UK) were placed and used to separate the donor and receptor chambers, just acting as physical support for the hydrogel and not as a permeation barrier. The membranes were boiled in ultra-purified water (Milli-Q^®^ water) for 10 min in order to hydrate them. Over the membrane, in the donator chamber, 0.025 g of each hydrogel was placed. The receptor chamber of the Franz diffusion cells was filled with degassed, ultra-purified water, which was maintained at a constant temperature of 32 ± 0.5 °C (to reproduce human skin temperature) through a thermostatic bath circulation. The experiment lasted for 30 min, this being the typical time of topical nanoclay/spring water hydrogels application. At the end of the experiments, the receptor aqueous phase was withdrawn and filtered through 0.45 μm single-use, sterile filters. Then, the elemental composition on each sample was assessed by ICP-MS, following the same protocol previously described for the pristine materials. All samples (six replicates) were subjected to in vitro release experiments 48 h after the preparation and after one month. During the experiment, the manipulation of different materials and instruments could contaminate the ultra-purified water of the Franz cell receptor chamber. To eliminate this error, blanks were also analyzed in order to monitor the elements coming from the materials and the ultra-purified water itself. Briefly, it consisted of analyzing the ultra-purified water that was placed in the receptor chamber of the Franz cell device. The concentration of the elements detected in the Milli-Q water (which was considered the blank, data not shown) were subtracted from the concentration detected in the receptor chamber. This way, it will be possible to discern the real number of elements exchanged/released from the semisolid formulation and, thus, able to establish contact with the patient skin.

### 2.3. Result Discussion and Interpretation Bases and Criteria

Different regulations could be used for the interpretation of the obtained results. In this paperwork, the discussion and interpretation of the results will be centered on the documents summarized in Table 1.

**Figure 1:**
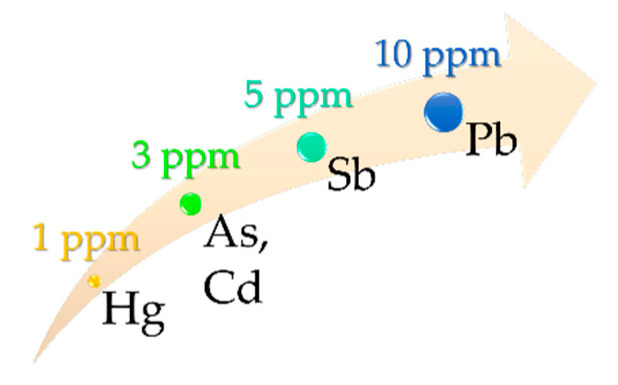
Acceptable limits for heavy metals in topical products [39].

The HC-SC guideline is focused on heavy metals (As, Pb, Cd, Hg, and Sb). Other metal elements such as Se, Ba, and Cr are considered less significant in terms of toxicity; therefore, no impurity limits are found for these elements in this document [1]. The Natural and Non-prescription Health Products Directorate (NNPHD) [39] is a guidance document intended to give support to stakeholders “in assuring that natural health products are produced in a high-quality manner”. NNPHD is focused on natural and non-prescription health products, which is the case of PS9, G30, and ALI ingredients and resultant hydrogels. Acceptable limits for As, Cd, Pb, Hg, Cr, and Sb elemental impurities are defined in this guide, including the limits for topical administration (Figure 1).

**Figure 2:**
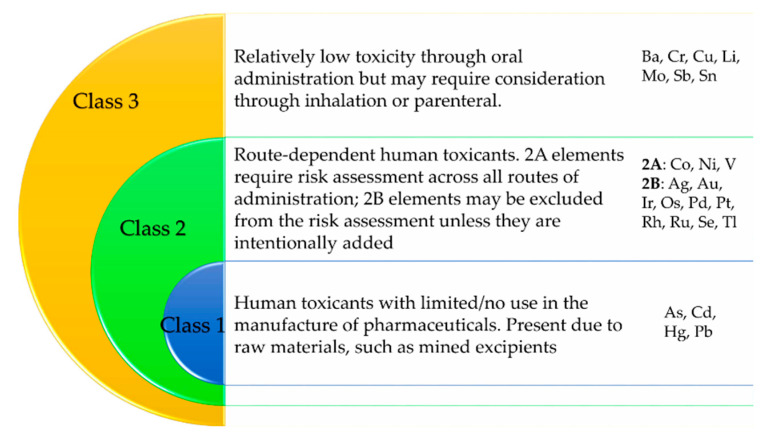
Element classification of the ICH Q3D(R1) guideline. Based on ICH Q3D(R1) [30].

The ICH guideline for Elemental Impurities Q3D(R1) refers to medicinal products and classifies elements into four groups based on their toxicity and likelihood of occurrence in these products (Figure 2). The fourth group called “other elements” includes elements for which Permitted Daily Exposure (PDE) limits have not been established. As a result of this, these elements have not been included in this manuscript. Regarding the most toxic elements, the Q3D(R1) guideline also reports the PDE limits by oral, parenteral, and inhalation administration routes [30]. PDE is the maximum acceptable intake of the elemental impurity per day. Although this guideline is not specific for cosmetics, the fact that it deals with elemental impurities of drugs (which are intended to reach the bloodstream) makes it useful to also ensure the safety of cosmetics.

Finally, the European Regulation EC 1223/2009, from all the documents and guidelines included in this study, is the most restricting one in terms of impurities present in cosmetics [3]. Only those elements considered safe and innocuous for the human being are allowed in cosmetics, while the rest of them are banned without any limit or maximum dose established. Furthermore, according to article 17 of this regulation, “the presence of traces of banned substances will only be allowed if they are technically inevitable and do not impair the safety of the cosmetic product”. In this regard, in vitro studies such as Franz cells could help to discern and discuss this point since they allow the study of the mobility of the elements present in the ingredients.

#### Dose of Nanoclay/Natural Spring Water Hydrogels

The toxicity of elemental impurities obviously depends on the administration route of the dosage form. Nanoclay hydrogels prepared in this study are intended to be topically applied over either the healthy or wounded skin of patients subjected to balneotherapy treatments. In thermal stations, natural or artificial clay-based/spring water formulations could be applied in different forms. The most common one includes the administration of semisolid systems at 45–50 °C on restricted body regions (mainly isolated joints) with 210 cm of thickness or in the form of total/partial baths. Most of these treatments usually last for 15 to 30 min [40,41,42]. The density of the hydrogels was obtained by the Minimum Square Method applied to experimental volume, and mass hydrogels measurements (R^2^ were >0.998 in both cases): ρALIPS9 = 1.0606 g/mL; ρALIG30 = 1.0992 g/mL. These data would be used to calculate safe doses of hydrogels in order to not exceed the PDE limits defined in the Guideline for Elemental Impurities [30] for each element. Moreover, despite the bioavailability of topically administered dosage forms hardly reaching 100%, in the discussion, we will systematically consider the maximum potential bioavailability in order to guarantee safe doses.

## 3. Results

### 3.1. Elemental Composition of Pristine Ingredients

The elemental composition of PS9, G30 nanoclays, and natural spring water (ALI) is summarized in Table 2. Below, the results will be discussed from the most innocuous to the most dangerous elements, as well as by the amount in which they were detected.

Regarding PS9 and G30, Ba, Cr, and Li (Class 3) and V (class 2A), were detected in remarkable amounts. The presence of Zr and Nd was also reported. Except for Li, the amount of the aforementioned elements was higher in G30 than in PS9, thus highlighting the presence of more impurities in G30. This statement is in agreement with the solid-state characterization of G30 made by García-Villén et al. [32]. The presence of hazardous elements such as Pb, As, and Cd in fibrous clay minerals have been reported as a common feature of natural deposits, though the present values are minimal with respect to previously reported levels [43].

With respect to the ALI spring water, the main hazardous impurities detected were Li and Ba, followed by Cr (class 3 elements in all cases). Therefore, the major elements detected (Table 2) belong to class 3 or class 2A [30], which indicates that they are elements whose presence in the raw materials should be borne in mind, though with relatively low toxicity. Additionally, the presence of Cr, Zr, and Nd is not allowed in cosmetics, according to the EC 1223/2009 [3]. From class 2B, only the presence of Se and Tl is banned in cosmetics, though the three of them were detected as traces (Table 2). Ni, which belongs to class 2A and its not allowed in cosmetics, was detected in significant amounts in G30, unlike PS9 and ALI.

Class 1 element group is formed by hazardous elements As, Cd, Hg, nd Pb (Figure 1), all of them prohibited according to EC 1223/2009 [3,30]. As, Cd, and Pb were similar to the ones reported for natural products used in cosmetics [8]. Unlike class 1 and EC 1223/2009, the NNHPD [39] specifies the acceptable limits for heavy metals in topical products (Figure 1), including Sb (class 3). All the aforementioned elements were below the limits established by the NNHPD.

The rest of the elements (from P onwards, Table 2) are not classified in Q3D(R1), thus not belonging to any specific group previously mentioned. Among them, Zr highlights due to the high amount present both in PS9 and G30 in comparison with the rest of the non-allowed elements.

### 3.2. In Vitro Release of Hazardous Elements from Hydrogels

The results obtained after the Franz cell studies regarding the release of the elements from nanoclay hydrogels at 48 h and after one month are summarized in Table 3.

Ba release was very variable between ALIPS9 and ALIG30, though the higher values detected in ALIG30 could be ascribed to higher Ba presence in the pristine material G30 in comparison with PS9 (Table 2). Cu mobility, which was higher in ALIG30 due to a higher amount in G30, significantly decreased after one month for both ALIG30 and ALIPS9.

The Li release was higher in ALIPS9 due to the higher Li levels in PS9 and maintained constant with time (no significant differences between 48h and 1 month). On the other hand, ALIG30 hydrogels showed a reduction in Li release as time passed. The highest Mo was found in ALIG30—48 h and significantly decreased after one month. On the other hand, ALIPS9 demonstrated a constant release of Mo through time. Sn, V, and Cd released from both hydrogels came from clay minerals since none of these elements were detected in ALI (Table 2). The V release increased with time, while Sn showed the opposite trend.

Heavy metals Hg, Pb, and Sb, though present in the pristine materials, were not released. Other not-allowed elements, such as Cr, Se, Tl, Ni, P, Be, Zr, Te, Nd, and Ta were neither release elements, which means that they do not pose any problem in terms of safety. On the other hand, Cd and As were slightly released, with higher results in the case of ALIG30 hydrogels. The absence of Cd in ALIPS9 is due to the extremely low amounts detected in PS9 and its absence in ALI. On the contrary, G30 possessed a higher amount of Cd (Table 2), which explains the release results (Table 3). In conclusion, Cd and As are the most crucial elements determining the safety of the hydrogels. It is worth mentioning that with respect to the As and Cd amounts and release, ALIPS9 hydrogel is considered the safest formulation.

## 4. Discussion

### 4.1. In Vitro Release of Elements: Safety Concerns and Doses

The toxicity of elemental impurities obviously depends on the administration route of the dosage form. The studied hydrogels are topically administered, and the bioavailability of a certain element hardly reached 100%. Tateo et al. [9], in previous studies regarding elemental percutaneous mobility, stated that the major part of the elements could cross the skin. Nonetheless, they reported that “none of these elements reaches concentrations so high as to represent hazardous conditions”. In the discussion, we systematically will consider a theoretical 100% bioavailability in order to guarantee safe doses in any case.

According to the results, the maximum amount of Ba released came from ALIG30, 48 h, and it counted for 8.3 μg/100 g of hydrogel. The oral PDE of barium was established as 730 μg/day [30]. If we consider the maximum mobility and a 100% bioavailability of Ba through the skin, the administration of ALIG30 and ALIPS9 hydrogels would be considered safe if doses are less than 8.79 kg hydrogel/day (Table 4). In view of the high amounts of hydrogels needed to pose a risk regarding Ba, it is possible to state that both ALIPS9 and ALIG30 are safe with respect to this element.

Among the possible adverse effects associated with Cu, allergic dermatitis is the most commonly experienced [44]. Safe amounts of hydrogels regarding Cu release (Table 3) have been calculated according to parenteral PDE (Table 4). In view of the results, hydrogels aged for one month could be considered safe in terms of allergenic copper effects, since its mobility practically disappears. Moreover, ALIPS9 would be more advisable than ALIG30; the amount of Cu being lower in the former one. The amount of Cu released from extemporaneous formulated hydrogels could limit their use in general baths, as the calculated safe dose (Table 4) should be lesser than two kilograms of hydrogel.

Li is of relatively low toxicity by the oral route. Is a common metal present in animal tissues and is used in certain kinds of treatments, such as bipolar disorder or depression, among others. Recently, Yuan et al. [45] prepared a sponge scaffold with LiCl and evaluated wound healing activity in vitro. The presence of Li reduced inflammation and improved angiogenesis, re-epithelialization, and expression of β-catenin. Seborrheic dermatitis is another skin disorder that has been addressed by Li as an active lithium gluconate/succinate, with successful results [46,47,48,49]. The main problem of Li is the narrow therapeutic margin it possesses [50,51]. Parenteral Li PDE was established to be 280 μg/day [30]. Considering that all the released Li would be able to reach the bloodstream once the hydrogel is applied, the administration of ≤ 1.37 kg hydrogel/day would guarantee safe doses of Li (below the parenteral PDE, Table 4).

Mo could be considered as an essential element since its deficiencies have been related to night-blindness, nausea, disorientation, coma, tachycardia, tachypnea, and other biochemical abnormalities [30]. Nonetheless, excessive accumulation of Mo could also produce toxicity, so its limits need to be controlled. In particular, Mo could be accumulated in the skin, bound to dermal collagen. The amount of Mo released from ALIPS9 was constant with time, while it significantly reduced after one month in ALIG30 (Table 3). Parenteral PDE levels of Mo are 1700 μg/day. Considering the highest released amount of Mo (ALIG30—48 h), and supposing 100% of bioavailability, 94 kg/day of hydrogels would be necessary to reach PDE limits (Table 4). In view of these calculations, it is possible to guarantee that ALIPS9 and ALIG30 are safe with respect to Mo levels.

Tin is an element widely used nowadays [52]. The PDE limits of Sn have been established since it has been reported to increase in vitro oxidative stress or DNA breakage [53]. In view of tin’s released amounts in ALIPS9 and ALIG30, its toxicity and PDE limits should be borne in mind. In particular, this element showed lower release from both hydrogels after one month of preparation. The inhalation and oral consumption of Sn are the main routes for Sn intoxication [54,55,56], thus meaning that the topical application of these hydrogels would be a safe administration route. In fact, in vitro cytotoxicity studies of these hydrogels were not shown to hinder normal dermal human fibroblast growth nor cell motility during in vitro wound healing [57].

Ni has been widely detected in cosmetic products, together with Co and Cr, among others [8,58]. The attention paid to Ni, Co, and Cr is based mainly on skin conditions, such as contact allergic dermatitis, itching, and edema, among others [59,60,61,62,63,64]. What is more, these elements can be solubilized by sweat during prolonged contact [60,65]. Pristine materials possessed higher amounts of Ni than Co. Nonetheless, no Ni and Cr mobility was detected in Franz cell tests, thus reducing the risk of contact skin alterations produced by ALIPS9 and ALIG30 hydrogels. The Co mobility in ALIPS9 increased with time, while in ALIG30, it maintained constant (Table 3). The application of young hydrogels (ALIPS9) would entail the lowest risk of skin allergies related to cobalt. Co is an integral component of vitamin B_12_, which means that it is essential for the human body. It is estimated that the average person receives about 11 μg Co/day with normal diet, and the parenteral PDE is established to be 5 μg/day [3]. Additionally, it has been demonstrated that Co is able to pass the skin [65,66], though its percutaneous absorption was found to be very low (0.0123 μg·cm^−2^·h^−1^). Time of hydrogels application in thermal stations takes at about 20–30 min, which is not enough time for all the mobile Co (Table 3) to cross the human skin, thus making the “hydrogel safe dose/day” (calculated assuming a 100% of bioavailability, Table 4) to be remarkably higher in real conditions.

V is a ubiquitous element in the human body, though no essential role has been found yet for this element. Although systemic toxicity of V has already been accepted, its deficiency has also proved to be problematic since it is associated to thyroid, glucose, and lipid metabolism malfunctions. It also participates in the regulation of several genes and has been demonstrated to influence cancer development, including skin cancers such as malignant melanoma [67,68]. V was a mobile element in both hydrogels prepared. No V was detected in the ALI, Franz cells results ascribable to pristine clay minerals composition (Table 2). In ALIG30, the mobility of V was higher due to the higher amount of this element in G30 in comparison with ALIPS9 (Table 3). Although antiproliferative properties of V ions have been found [68], neither ALIPS9 nor ALIG30 hydrogels impaired normal human fibroblasts in vitro proliferation, according to previous studies [57].

As is a forbidden element in cosmetics [3]. Arsenic mobility is reported to be very low in both hydrogels. It maintains constant through time in ALIG30, while it increases in ALIPS9 after one month. This element is very ubiquitous in the environment, so it is expected to be present in natural ingredients such as clay minerals. This element is classified in the category 1A in view of its carcinogenicity, as reported in the European Regulation EC 1272/2008 [69]. For greater clarity, substances belonging to the 1A category are known to have carcinogenic potential to humans. It possesses a pronounced affinity for skin and keratinizing structures, although it does not act as a sensitizer due to poor skin penetrating ability. According to article 1 of the EC 1223/2009, “prohibited substances should be acceptable at trace levels only if they are technologically inevitable with correct manufacturing processes and provided that the product is safe”. Assuming 100% bioavailability and using parenteral PDE levels, the maximum dose of hydrogels that could be used without exceeding these levels is 3.75 kg·day^−1^ (Table 4) [30]. Moreover, the maximum mobility of As is far less than the maximum permissible concentration of inorganic As in drinking water (10 μg/L [70,71] vs. 4.24 μg/L in ALIPS9 one month).

Cd is another element whose presence in cosmetics is forbidden. Cd did not show mobility from ALIPS9. Higher amounts of Cd in G30 justified the higher mobility of this element in ALIG30. The low release of Cd from fibrous clay minerals is in agreement with previous studies, which reported irreversible interaction between Cd and the solid phase, thus hindering the mobility of this element [72]. Regarding toxicity, Cd is classified in the category 2 (suspected human carcinogen). It can accumulate in the skin, having deleterious effects on this organ, as recently demonstrated by an in vivo study [73]. Nonetheless, the in vitro percutaneous bioavailability of cadmium chloride salt tested through human skin was determined to be among 0.07% (from water) and 0.01% (from soil) [74].

Other heavy metals such as Pb and Sb, though present in the pristine materials (Table 2), were not mobile (Table 3). Hg and Au were not detected in the pristine materials, and their absence after Franz cell diffusion guaranteed the absence of contamination during preparation, conservation, packaging, and manipulation of the hydrogels (art. 17 of the EC 1223/2009 [3]). Additionally, the cytotoxicity of these hydrogels has been previously tested in vitro [57], obtaining very positive results, which supports the hypothesis of product safety.

### 4.2. Mobility of Hazardous Elements

The presence in a product of an elemental impurity will have safety concerns only once released, with different mechanisms underlying the release of each particular element, mainly depending on its position in the structure of the hydrogel components. Nevertheless, it is possible to calculate a parameter of general comparative interest; mobility of the elements from the dosage form. The mobility of an element could be calculated as the ratio between the element content/element released.

**Figure 3:**
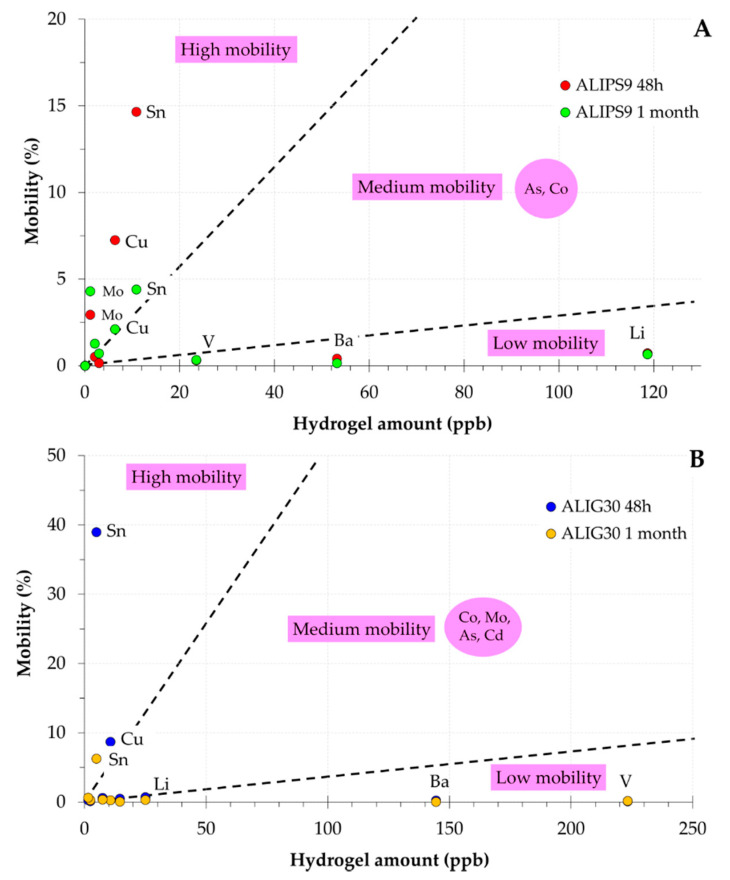
Element mobility (%) versus the total amount of element in ALIPS9 (**A**) and ALIG30 (**B**). Only mobile elements are shown.

**Figure 4:**
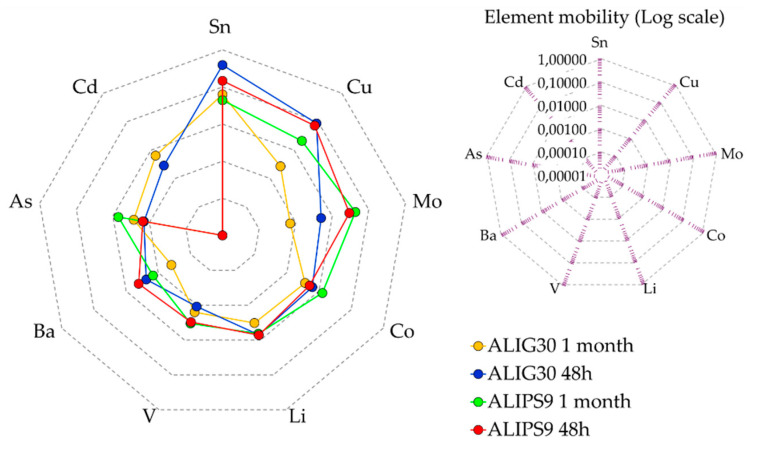
Spider diagram of element mobility. Only mobile elements are shown. The logarithmic scale was used, though not included in the spider diagram for simplicity and clarity.

In view of the previously shown results, the mobility of hazardous elements from ALIPS9 and ALIG30 hydrogels was minimal in the major part of the cases (Figure 3). These results could be explained by the high adsorption capacity of palygorskite and sepiolite clay minerals, their low cation exchange capacity, and the gel network of hydrogels. The mobility of tracers has been explained due to the formation of inner-sphere complexes with clay and other associated mineral surfaces [75,76,77]. It also seems clear that different ionic equilibriums are established through time in both hydrogels (Figure 4). For instance, Co, Ba, and Sn reduce their mobility while As and Cd increase it, thus demonstrating that elements established different equilibriums within the hydrogel.

Sn showed to be the most mobile element, followed by Cu (Figure 3). Sn was not detected in ALI, which indicates that the release of this element came from PS9 and G30. On the other hand, Cu was detected in the three ingredients. In ALIPS9, the third element with significant mobility was Mo (Figure 3A). Molybdenum was also detected as an element with remarkable mobility in ALIPS9, while the mobility in ALIG30 was significantly smaller. The differences in Mo amounts released (Table 3) could be ascribed to the differential solid-liquid equilibrium established within both hydrogels since both PS9 and G30 presented the same amount of this element (Table 2). The reduction of Mo mobility in ALIG30 indicates that the G30 clay mineral has a better ability to retain the Mo in ALI.

Li, Ba, V, and Cd were the elements with the smaller mobility. V was not detected in ALI, which means that the released amount was due to the clay minerals. V and Li did not participate in the solid-liquid equilibrium established between solid and liquid phases since their mobility was constant with time. Although Ba was an element with minimal mobility, it reduced with time in both hydrogels. The fact that G30 showed higher Ba amount than PS9 together with the fact that Ba mobility in this hydrogel was smaller than in ALIPS9 indicates that it is a structural element of G30. Moreover, the similar release of Ba from ALIPS9 and ALIG30 means that, probably, the major part of the Ba released came from ALI instead of PS9 and G30.

Cd and As were slightly mobile, with higher results in the case of ALIG30 hydrogels. The absence of Cd mobility in ALIPS9 was due to the extremely low amounts detected in PS9 and its absence in ALI. On the contrary, G30 possessed higher amounts of Cd (Table 2), which explains the mobility results (Table 3). Cd and As were the most crucial elements determining the safety of the hydrogels. It is worth to mention that, with respect to the As and Cd amounts and mobility, ALIPS9 hydrogels could be considered the safest formulation.

The mobility of Sn, Cu, Mo, and Ba from hydrogels reduced as time passed. The mobility reduction could be explained by the irreversible adsorption of the elements by PS9 and G30. In fact, fibrous clay minerals have been proposed as environmental remediation ingredients and wastewater treatments aiming to eliminate heavy metals with promising results [78,79,80,81].

The spider diagram (Figure 4) clearly shows that, as a general trend, palygorskite hydrogels reduced the mobility of hazardous elements with time (with a reduction in area), whereas this was not obvious for sepiolite hydrogels.

Finally, previous studies on elemental mobility from clay minerals, spring waters, and thermal muds have reported that the amount of released elements highly depends on their concentration in spring water, the release of elements from the solid phase being negligible [9]. Nonetheless, in view of the results of the present study suggest that solid-phase composition did play a crucial role. This discordance could be due to the high structuration of the present hydrogels.

At this point of the study, and looking at the safe dose calculations (Table 4), the way to guarantee the absence of any intoxication risk would be to apply the hydrogels locally (over restricted areas of the skin, wounds, joints, etc. That is, hydrogel bath treatment should be avoided if potentially toxic doses of the elemental impurities want to be minimized. Nonetheless, bioavailability and percutaneous permeation studies would be highly useful and valuable to establish safe usage guidelines for these formulations.

## 5. Conclusions

Elemental impurities in medicinal products have to be controlled within safety limits with different guidelines and normatives being useful from a pharmaceutical quality perspective. The essential role of clay minerals in drug products and cosmetics is widely known. Nanoclay/natural spring water hydrogels have been prepared by mixing a sepiolite and a palygorskite with local spring water (Alicún de las Torres, Granada, Spain). Clay hydrogels are traditionally used in balneotherapy or as natural cosmetics (masks, shampoos, etc.). Since these formulations are intended to establish an intimate contact with the skin (either healthy, sensitive, or damaged skin) their composition is of high importance in terms of safety. In this study, special attention has been paid to the presence of heavy metals and other hazardous elements. As expected, pristine materials possessed a wide variety of hazardous elements such as Cd, Pb, or P, among others. Since these elements are specifically forbidden in cosmetics according to the European Regulation (EC 1223/2009), pristine materials do not accomplish cosmetic regulations on their own. Nonetheless, the presence of certain substances in a cosmetic does not imply that they are able to be absorbed or enter in contact with the skin. In order to discern the potential bioavailability of these elements, their mobility was evaluated by using Franz cells in vitro tests. Among all the specifically forbidden elements in cosmetics, only As and Cd were detected as mobile, though in very low amounts. Their mobility was so low that, taking into account the corresponding PDE for the parenteral route and assuming 100% of bioavailability through the skin, the calculated safe doses were approximately 1 kg of hydrogel per day. In conclusion, the present study demonstrates that the composition and nature of the solid phases of the hydrogel determine the mobility of the elements. Legally speaking, the mobility of As and Cd could hinder the authorization of ALIPS9 and ALIG30 hydrogels as cosmetic products. Nonetheless, there is no sufficient evidence to confirm that the presence of these elements is detrimental to their safety and, though further studies are still necessary, ALIPS9 and ALIG30 hydrogels could be used in practice. Finally, it is worth to mention that, despite that ALIG30 showed higher ability to reduce the elements mobility, the ALIPS9 hydrogel would be easier to authorize as a medicine or cosmetic, since the mobility of As and Cd in this hydrogel was minimum or absent. Future perspectives of this particular study include the assessment of the percutaneous mobility of the elements (bioavailability) both in vitro and in vivo. These kinds of studies would help to better define the best techniques to apply fibrous clay-based hydrogels to maximize benefits by minimizing the risks.

## Figures and Tables

**Table 1:** Documents used during the interpretation and discussion of the obtained results.

Type of Document	Region	Year	Ref
Regulation of the European Parliament and Council of the European Union on cosmetic products (EC 1223/2009)	EU	2009	[3]
Guidance on Heavy Metal Impurities in Cosmetics (HC-SC)	Canada	2012	[1]
Quality of Natural Health Products Guide - Natural and Non-prescription Health Products Directorate (NNPHD)	Canada	2015	[39]
Guideline for Elemental Impurities Q3D(R1)	EU	2019	[30]

**Table 2:** Elemental composition of pristine ingredients (PS9 and G30 nanoclays and ALI spring water) determined by ICP-MS. The elements are classified depending on regulations [3,30]. “ND” stands for “Not detected”.

Element	PS9 (ppm)	G30 (ppm)	ALI (ppb)	Comments
Ba	56.2	144.8	18.8	Class 3 in Q3D(R1) [30]; Not listed as element in EC 1223/2009 [3]
Cr	14.0	391.8	4.3	Class 3 in Q3D(R1) [30]; Not allowed in EC 1223/2009 [3]
Cu	8.1	11.3	2.5	Class 3 in Q3D(R1) [30]; Allowed in EC 1223/2009 [3]
Li	149.0	30.4	244.2	Class 3 in Q3D(R1) [30]; Not listed in EC 1223/2009 [3]
Mo	0.2	0.2	3.8	
Sb	0.3	2.1	0.1	Class 3 in Q3D(R1) [30]; Not allowed in EC 1223/2009 [3]
Sn	10.8	3.3	ND	Class 3 in Q3D(R1) [30]; Not listed in EC 1223/2009 [3]
Ag	0.04	0.2	0.1	Class 2B in Q3D(R1) [30]; Allowed in EC 1223/2009 [3]
Au	ND	ND	ND	
Ir	0.2	0.9	ND	Class 2B in Q3D(R1) [30]; Not listed in EC 1223/2009 [3]
Se	0.9	1.5	2.3	Class 2B in Q3D(R1) [30]; Not allowed in EC 1223/2009 [3]
Tl	0.2	0.1	0.1	
Co	2.3	7.4	0.4	Class 2A in Q3D(R1) [30]; Not listed in EC 1223/2009 [3]
Ni	3.7	50.7	9.4	Class 2A in Q3D(R1) [30]; Not allowed in EC 1223/2009 [3]
V	24.9	249.1	ND	Class 2A in Q3D(R1) [30]; Not listed in EC 1223/2009 [3]
As	2.0	1.3	0.2	Class 1 in Q3D(R1) [30]; Not allowed in EC 1223/2009 [3]
Cd	0.02	1.5	ND	
Hg	ND	ND	ND	
Pb	3.2	4.1	ND	
P	0.3	8.5	0.1	Not allowed in EC 1223/2009 [3]
Be	1.8	3.6	0.01	
Zr	21.5	50.3	0.2	
Te	ND	ND	ND	
Nd	8.0	30.2	ND	
Ta	0.9	0.7	0.005	

**Table 3:** Mobility of hazardous elements after Franz diffusion cell tests. The concentrations are expressed in μg/100 g of hydrogel. Elements have been placed in the same order as in Table 2. Mean values ± s.e. (*n* = 6). “ND” stands for “not detected”.

Elements	ALIPS9		ALIG30	
	48h	1 Month	48h	1 Month
Ba	5.6 ± 1.55	1.8 ± 0.934	8.3 ± 0.944	1.2 ± 0.360
Cr	ND		ND	
Cu	10.8 ± 3.293	3.6 ± 2.17	20.6 ± 3.725	0.91 ± 0.608
Li	20.5 ± 3.293	17.7 ± 3.214	4.3 ± 0.362	1.7 ± 0.379
Mo	0.7 ± 0.095	0.61 ± 0.125	1.8 ± 0.0572	0.21 ± 0.123
Sb	ND		ND	
Sn	28.7 ± 8.232	10.4 ± 2.138	50.4 ± 4.866	6.5 ± 1.945
Ag, Au, Ir, Se, Tl	ND		ND	
Co	0.26 ± 0.206	0.6 ± 0.093	1.1 ± 0.660	0.58 ± 0.237
Ni	ND		ND	
V	1.7 ± 0.310	1.8 ± 0.492	5.9 ± 0.306	7.5 ± 0.315
As	0.1 ± 0.010	0.4 ± 0.039	0.08 ± 0.050	0.1 ± 0.063
Cd	ND	ND	0.1 ± 0.064	0.2 ± 0.0087
Hg, Pb	ND		ND	
P, Be, Zr, Te, Nd, Ta				

**Table 4:** Theoretical safe doses of ALIPS9 and ALIG30 hydrogels based on elements with defined parenteral Permitted Daily Exposure (PDE) levels. Calculations have been made by using the higher mobility value reported by Franz cells (either ALIPS9 or ALIG30). Additionally, safety doses are calculated assuming a theoretical dermal bioavailability of 100%.

Element	PDE_parent_ Limits [29]	Maximum Release Detected	Hydrogel Safe Dose/Day
Ba	730 μg/day	8.3 μg/100 g (ALIPS9–1 month)	≤8.79 kg
Cu	340 μg/day	20.6 μg/100 g (ALIG30–48 h)	≤1.65 kg
Li	280 μg/day	20.5 μg/100 g (ALIPS9–48 h)	≤1.37 kg
Mo	1700 μg/day	1.8 μg/100 g (ALIG30–48 h)	≤94.4 kg
Sn	640 μg/day	50.4 μg/100 g (ALIG30–48 h)	≤1.27 kg
Co	5 μg/day	1.1 μg/100 g (ALIG30–48 h)	≤454 g
V	12 μg/day	7.5 μg/100 g (ALIG30–1 month)	≤160 g
As	15 μg/day	0.4 μg/100 g (ALIPS9–1 month)	≤3.75 kg
Cd	1.7 μg/day	0.2 μg/100 g (ALIG30–1 month)	≤850 g
